# Three-Dimensional Image Transmission of Integral Imaging through Wireless MIMO Channel

**DOI:** 10.3390/s23136154

**Published:** 2023-07-04

**Authors:** Seung-Chan Lim, Myungjin Cho

**Affiliations:** School of ICT, Robotics and Mechanical Engineering, Institute of Information and Telecommunication Convergence (IITC), Hankyong National University, 327 Chungang-ro, Anseong 17579, Kyonggi-do, Republic of Korea; sclim@hknu.ac.kr

**Keywords:** multiple-input multiple-output (MIMO), three-dimensional integral imaging, volumetric computational reconstruction (VCR)

## Abstract

For the reconstruction of high-resolution 3D digital content in integral imaging, an efficient wireless 3D image transmission system is required to convey a large number of elemental images without a communication bottleneck. To support a high transmission rate, we herein propose a novel wireless three-dimensional (3D) image transmission and reception strategy based on the multiple-input multiple-output (MIMO) technique. By exploiting the spatial multiplexing capability, multiple elemental images are transmitted simultaneously through the wireless MIMO channel, and recovered with a linear receiver such as matched filter, zero forcing, or minimum mean squared error combiners. Using the recovered elemental images, a 3D image can be reconstructed using volumetric computational reconstruction (VCR) with non-uniform shifting pixels. Although the received elemental images are corrupted by the wireless channel and inter-stream interference, the averaging effect of the VCR can improve the visual quality of the reconstructed 3D images. The numerical results validate that the proposed system can achieve excellent 3D reconstruction performance in terms of the visual quality and peak sidelobe ratio though a large number of elemental images are transmitted simultaneously over the wireless MIMO channel.

## 1. Introduction

Recently, the transmission technique of three-dimensional (3D) digital content has become important in various applications such as virtual reality (VR), augmented reality (AR), 3D TV, the metaverse, and so on. Such 3D digital content can be generated by various methods such as stereoscope, integral imaging, holography, etc. Especially, integral imaging which was first proposed by G. Lippmann [1], has been studied by many researchers. It can obtain 3D images using a lenslet array or camera array and provide 3D images without a coherent light source such as a laser and a special viewing device. In addition, it can provide 3D images with a full parallax and continuous viewing points passively. However, it has several drawbacks: low viewing resolution, narrow viewing angle, and shallow depth of field.

Many studies have been reported to overcome the drawbacks of the integral imaging [2,3,4,5,6,7,8,9,10,11,12,13]. To improve the viewing resolution of 3D images, the moving array lenslet technique (MALT) [6] was proposed. It uses the afterimage effect of human eyes. However, it may not record a 3D dynamic scene because of the mechanical movement of the lenslet array. Synthetic aperture integral imaging (SAII) [7], which uses a camera array, can improve the viewing resolution of 3D images since each elemental image has the same resolution as the image sensor. In [8], the SAII is further merged with an axially distributed sensing method to improve the lateral and longitudinal resolutions of 3D objects. However, it is not cost-effective and there exists a synchronization problem between cameras. The viewing angle of 3D images in integral imaging can be determined by the focal length of the lenslet and pitch between the elemental images. To enhance the viewing angle, integral imaging with a low fill factor, which can increase the pitch between cameras, was proposed [9]. However, the intensity of the 3D images is reduced due to the low fill factor of the elemental images. The depth of field of 3D images in integral imaging depends on the characteristics of lenslet such as the focal length of the lenslet, distance between the lenslet and image sensor, and the diffraction limit. To solve this problem, depth-priority integral imaging was proposed [10]. It can provide the plane wave of each pixel by setting the distance between the lenslet and image sensor as the focal length of the lenslet. However, the viewing resolution of 3D images is degraded since the spot size of 3D images in this method is the same as the lenslet size.

Volumetric computational reconstruction (VCR) [11,12] of integral imaging may solve these drawbacks. Using high-resolution elemental images recorded by SAII, the VCR can reconstruct 3D images at the desired reconstruction depth by back-projecting elemental images through a virtual pinhole array, overlapping each other, and averaging them. Thus, it can provide 3D slicing images at various reconstruction depths, and suppress the noise of 3D images by its averaging effect. By introducing an adjustable parameter to generate a group of 3D images, a four-dimensional image structure can be generated by the computational reconstruction method in [13]. However, all of the aforementioned studies regarding integral imaging may suffer from space limitations, because the pickup and reconstruction process must be performed in the same place and cannot be implemented apart. In fact, wireless 3D image transmission is readily implementable by performing the SAII and VCR at the transmitter and receiver, respectively. Nonetheless, a sophisticated system design is required to transmit a large number of high-resolution 2D elemental images through the wireless channel and display a 3D image with a high reconstruction performance while overcoming wireless impairments [14,15,16,17,18].

To this end, in this paper, we propose a novel 3D image wireless transmission system with the aid of the multiple-input multiple-output (MIMO) technique, which enables us to achieve a high transmission rate in wireless communication. In the proposed wireless 3D image transmission system, both the transmitter and receiver are equipped with multiple antennas to exploit the spatial multiplexing capability of a MIMO channel [19,20]. At the transmitter, individual 2D elemental images, picked up by multiple sensors, are converted to distinct data streams, and transmitted concurrently through the corresponding antennas. For the successful 3D image reconstruction, the receiver needs to recover multiple elemental images by overcoming the inter-stream interference caused by the simultaneous transmission. Accordingly, we develop a simple receiver architecture by applying various linear combiners, such as matched filter (MF), zero forcing (ZF), and minimum mean squared error (MMSE) [21]. Finally, using the recovered elemental images, the 3D image is reconstructed by applying the VCR with non-uniform shifting pixels. Based on the optical experiments, we verify that the proposed system is feasible for reconstructing 3D images and achieving sufficient peak sidelobe ratio (PSR) performance at the desired reconstruction depth. Furthermore, we find that the application of the MF combiner can achieve notable 3D reconstruction performance with practical computational complexity with the aid of the averaging effect of the VCR, despite severely poor wireless communication performance.

This paper is organized as follows. In Section 2, we present the related work briefly. Then, we describe the transmission of 3D integral imaging over a wireless MIMO channel in Section 3. To prove the proposed system, we show the experimental results in Section 4. Finally, we conclude with a summary in Section 5.

*Notation*: For a vector, superscript ${}^{T}$ denotes the transpose operation. For a matrix, superscripts ${}^{†}$ and ${}^{-1}$ represent the complex conjugate transpose and inverse operations, respectively. For a random variable, E stands for the expectation, i.e., statistical mean. An identity matrix with size *N* is denoted by $I_{N}$.

## 2. Related Work

### 2.1. Integral Imaging

**Figure 1:**
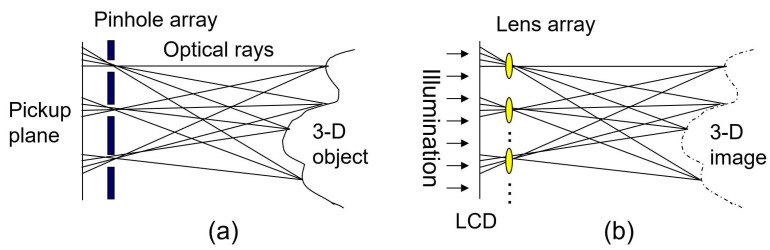
Integral imaging. (**a**) Pickup and (**b**) reconstruction.

Figure 1 illustrates the concept of integral imaging. Integral imaging consists of two processes; pickup and reconstruction. In the pickup process, as depicted in Figure 1a, 3D information of objects can be recorded by a pinhole or lenslet array. In fact, this 3D information has a different perspective from 3D objects. In addition, the recorded different perspectives are referred to as the elemental images. Then, in the reconstruction process, a 3D image can be reconstructed or displayed by illuminating these elemental images through the homogeneous pinhole or lenslet array used in the pickup process, as illustrated in Figure 1b. However, as shown in Figure 1b, the 3D image has reverse depth information, which is called the 3D pseudoscopic real image. To solve this problem, each elemental image is rotated by 180° [2]. As a result, the 3D orthoscopic virtual image can be displayed. In addition, the resolution of the 3D image is degraded by the number of pinholes or lenslets in the integral imaging. Therefore, a different pickup process is required to enhance the resolution of the 3D image.

**Figure 2:**
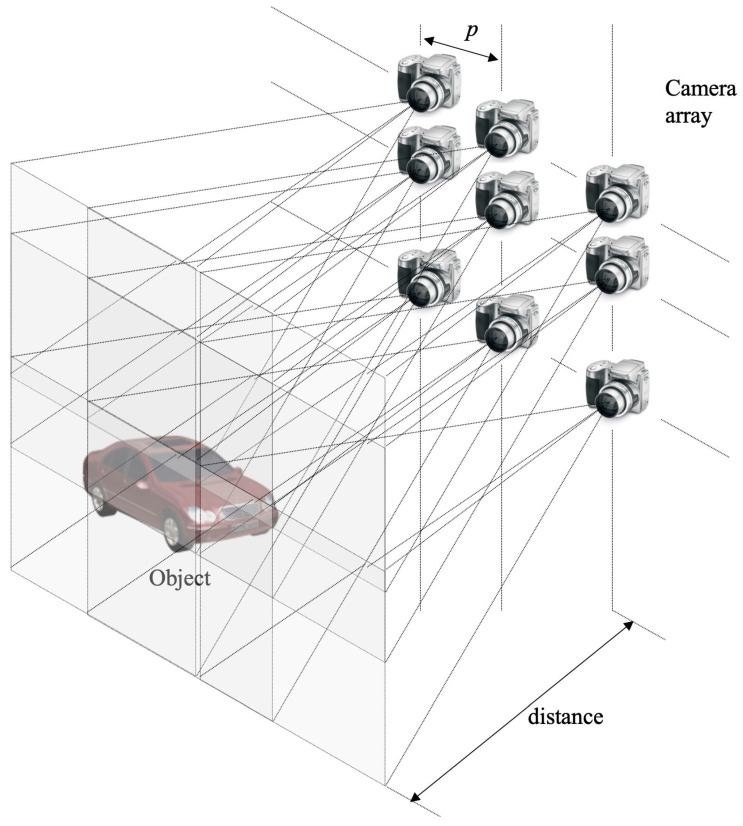
Synthetic aperture integral imaging.

Synthetic aperture integral imaging (SAII) [7] can improve the viewing resolution of a 3D image, as depicted in Figure 2. In SAII, a camera array is used to record the high-resolution elemental images instead of a pinhole or lenslet array. Thus, each elemental image has more pixels. This means that the lateral and longitudinal resolutions of 3D image can be improved. In addition, more accurate depth information can be obtained than through lenslet-array-based integral imaging since the pitch between the cameras (*p*) can be adjusted. Therefore, in this paper, we use SAII for the pickup process of integral imaging. However, optical reconstruction (i.e., display) may not be implemented since each elemental image has more pixels than the display panel. To reconstruct a high-resolution 3D image, a computational reconstruction technique in integral imaging is required.

**Figure 3:**
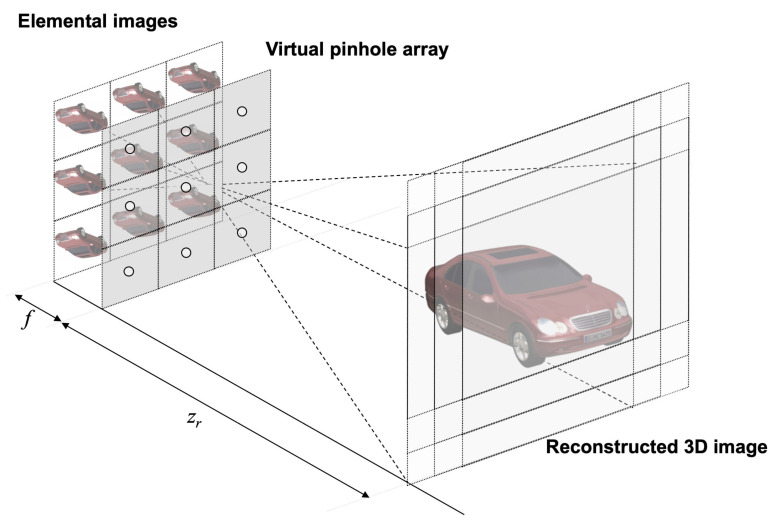
Volumetric computational reconstruction.

**Figure 4:**
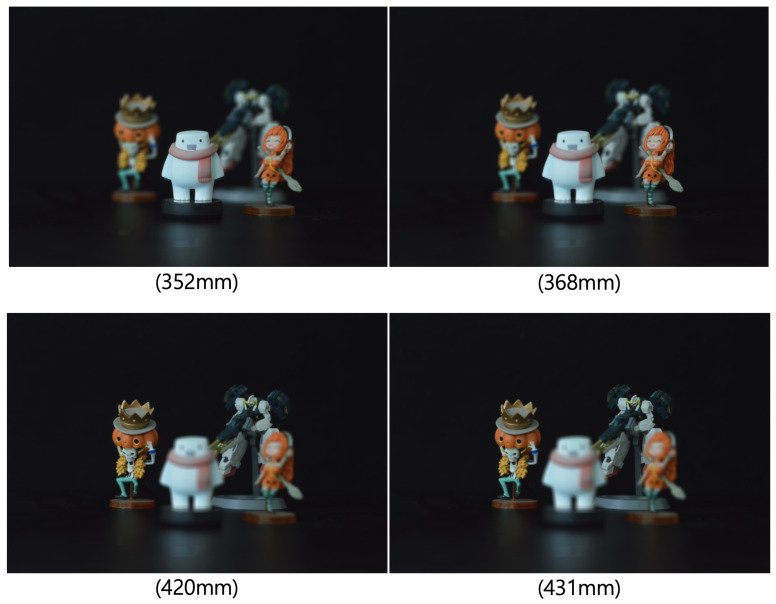
Reconstructed 3D images at various reconstruction depths.

Volumetric computational reconstruction (VCR) [11], as illustrated in Figure 3, has been proposed to reconstruct high-resolution 3D images. In VCR, each elemental image is back-projected through a virtual pinhole array on the reconstruction plane, where the distance between the elemental images and the virtual pinhole array is the focal length of the camera lens (*f*) in SAII and the reconstruction depth is the distance between the virtual pinhole array and reconstruction plane ($z_{r}$). On the reconstruction plane, each elemental image is overlapped with the shifting pixels, as expressed in Equation (1).

(1) $$S_{x}\left(k\right)=\left\lfloor \frac{N_{x}fp_{x}}{c_{x}z_{r}}\left(k-1\right)\right\rceil ,\;S_{y}\left(l\right)=\left\lfloor \frac{N_{y}fp_{y}}{c_{y}z_{r}}\left(l-1\right)\right\rceil$$

where $S_{x}\left(k\right)$, $S_{y}\left(l\right)$ are the shifting pixels, $N_{x}$, $N_{y}$ are the number of pixels, *f* is the focal length of the camera in SAII, $p_{x}$, $p_{y}$ is the pitch between the cameras in the x and y directions, $c_{x}$, $c_{y}$ is the sensor size in the x and y directions, and *k*, *l* is the index of the elemental images in the x and y directions. Thus, the 3D image can be reconstructed by the following:

(2) $$I\left(x,y,z_{r}\right)=\frac{1}{O(x,y,z_{r})}\sum_{k=1}^{K}\sum_{l=1}^{L}I_{kl}\left(x+S_{x}\left(k\right),y+S_{y}\left(l\right)\right)$$

where $O(x,y,z_{r})$ is the overlapping matrix at $z_{r}$, $I_{kl}$ is the elemental image of the *k*th column and *l*th row, and *K*, *L* are the total index of the elemental images in the *x* and *y* directions. As the reconstruction depth changes, different sliced 3D images can be generated by Equation (2). Figure 4 shows the reconstruction results using Equation (2). It is noticed that objects are focused at their own depths and they are blurred at other depths. In addition, VCR can enhance the visual quality of 3D reconstructed image when there is a lot of noise because VCR utilizes the average value of the overlapped elemental images.

As mentioned above, integral imaging can generate 3D digital content using elemental images with SAII and VCR. However, to broadcast this 3D digital content to multiple users, a new transmission technique is required, since the data amount of elemental images is extremely large.

### 2.2. MIMO Technique

The MIMO technique enables technology to enhance the performance of modern wireless communications [22,23]. In MIMO systems, multiple antennas are deployed at the transmitter and receiver to exploit the spatial multiplexing capability of the MIMO channels [19,20]. Theoretically, the application of the MIMO technique can increase the transmission rate proportionally to the minimum number of the antennas deployed at the transmitter and receiver [21]. To achieve a high transmission rate, independent data streams are transmitted simultaneously through multiple antennas. After passing over the wireless MIMO channel, independent data streams are multiplexed at multiple receive antennas. Due to the spatial multiplexing, the receiver needs to overcome the inter-stream interference to recover the transmitted data streams successfully. In this respect, a proper reception technique is required to improve the communication performance.

Although the joint maximum likelihood (ML) detection is optimal in terms of the theoretical capacity achievement of the MIMO channel [24], the computational complexity increases exponentially with respect to the number of data streams. Alternatively, the receiver can employ a linear combiner to convert the joint detection problem into several detection problems with low complexity by separating the multiplexed data stream into individual data streams. The MF combiner is known to be optimal for achieving the maximum received signal-to-noise ratio (SNR) [25]. However, the performance can be deteriorated severely for an interference dominant regime since the MF combiner cannot remove the inter-stream interference completely. The ZF is a widely used combining technique for nulling the inter-stream interference [26]. Although the ZF combiner is capable of removing the interference completely, the performance can be degraded due to the noise boosting effect, for a low-SNR regime in particular. On the other hand, the MMSE combiner can compensate the disadvantages of the MF and ZF [27]. The MMSE combiner achieves the maximum signal-to-interference-plus-noise ratio (SINR), meaning that it is an optimal scheme in the interference regime. As is conventionally reported in wireless communication theory, ZF and MMSE outperform MF for MIMO communication systems with a moderate number of antennas [21,28], but they require higher computational complexity due to the matrix inversion operation.

## 3. Wireless 3D Image Transmission System over MIMO Channel

### 3.1. Wireless 3D Image Transmission System

**Figure 5:**
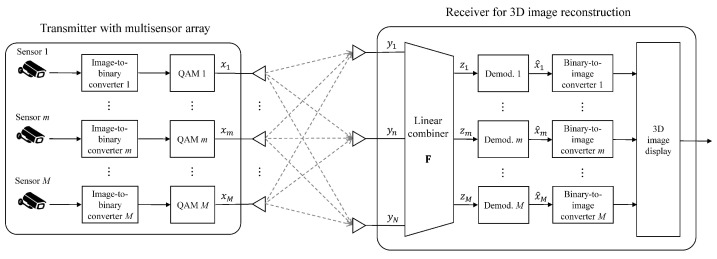
Wireless 3D transmission system, in which 2D elemental images are transmitted and received over an N×M MIMO channel.

In Figure 5, we illustrate the proposed wireless 3D transmission system, in which 2D elemental images, captured by *M* multiple sensors, are simultaneously transmitted to the receiver for the 3D image reconstruction. For the transmission and reception of elemental images, the transmitter and receiver are deployed with *M* and *N* antennas, respectively, where N≥M. Let $H\in C^{N\times M}$ be a Rayleigh fading channel matrix, in which each entry follows a complex Gaussian distribution with zero mean and unit variance. The fading channel is assumed to be static during a 3D image transmission, while the channel independently varies after a complete transmission. Moreover, we assume that the receiver perfectly obtains channel state information (CSI) by applying proper estimation techniques.

At the transmitter, *M* elemental images are picked up by the multiple sensors, and transmitted through the corresponding antennas. For m∈{1,…,M}, the *m*th image is converted to a binary sequence. Let Q be a constellation set of a quadrature amplitude modulation (QAM), where |Q|=Q. By applying the QAM, every $\log_{2}Q$ bits in the binary sequence are mapped to a complex symbol. For each time instance, let $x_{m}$ be a modulated symbol of the *m*th elemental image, where the average symbol energy is set to 1/M for equal power allocation over all antennas (i.e., $E[|x_{m}{|}^{2}]=1/M,\forall m$). A transmitted vector is defined as $x={\left[x_{1},\ldots ,x_{M}\right]}^{T}\in C^{M}$, in which the component $x_{m}$ is transmitted through the *m*th transmit antenna.

To successfully reconstruct the 3D image, the receiver needs to reliably recover the elemental images by overcoming the inter-stream interference arising from the simultaneous transmission of *M* streams. For each time instance, the received vector, denoted by $y={\left[y_{1},\ldots ,y_{N}\right]}^{T}\in C^{N}$, is written as

(3) y=Hx+v.

Here, $v\in C^{N}$ is an additive white Gaussian noise (AWGN) vector, where each component is an independent and identically distributed Gaussian random variable with zero mean and variance $\sigma^{2}$. With respect to the noise variance, the operating SNR is defined as $\rho =1/\sigma^{2}$. By assuming the perfect CSI, the receiver uses a linear combiner, denoted by $F\in C^{M\times N}$, to separate the received signal in Equation (3) into *M* streams. We consider three conventional combiners MF, ZF, and MMSE, which are defined as follows [21]:

(4) $$F=\left\{\begin{array}{cc} \frac{1}{N}H^{†},\; & \mathrm{for}\;\mathrm{MF},\\ {\left(H^{†}H\right)}^{-1}H^{†},\; & \mathrm{for}\;\mathrm{ZF},\\ {\left(H^{†}H+\frac{1}{\rho }I_{M}\right)}^{-1}H^{†},\; & \mathrm{for}\;\mathrm{MMSE}. \end{array}\right.$$

Multiplying the combining matrix F by the received vector yields

(5) z=Gx+w,

where the combined vector, channel, and noise are represented as $z=\mathrm{Fy}\in C^{M}$, $G=\mathrm{FH}\in C^{M\times M}$, and $w=\mathrm{Fv}\in C^{M}$, respectively. For m∈{1,…,M}, the *m*th component of the combined vector, denoted by $z_{m}$, is represented as

(6) $$z_{m}=g_{m,m}x_{m}+\sum_{i=1,\;i\neq m}^{M}g_{i,m}x_{i}+w_{m},$$

where $g_{i,j}$ is an entry corresponding to the *i*th row and *j*th column of G, and $w_{m}$ is the *m*th component of w. By treating the interference term $\sum_{i\neq m}g_{i,m}x_{i}$ in Equation (6) as noise, applying the ML detection leads to the estimation of the transmitted symbol, denoted by ${\hat{x}}_{m}$, as

(7) $${\hat{x}}_{m}=\underset{x_{m}\in Q}{\arg\max}{\left|z_{m}-g_{m,m}x_{m}\right|}^{2},$$

for all m∈{1,…,M}.

Because a binary sequence is readily obtained from ${\hat{x}}_{m}$’s for all time instances, the receiver is capable of estimating the *m*th elemental image by a binary-to-image conversion. Therefore, based on the process described in Section 2.1, the receiver can finally carry out the 3D reconstruction using *M* estimated elemental images which are transmitted over the N×M MIMO channel. Although the estimated 2D elemental images are corrupted due to the simultaneous transmission, a 3D image can be reconstructed with sufficient quality since the application of the VCR in (2) averages out statistically uncorrelated inter-stream interferences.

### 3.2. Discussion on the Linear Combiners

It is worth noting that we only need a matrix multiplication for the MF combining, which requires a computational complexity of $O(M^{2})$. Meanwhile, the implementation of the ZF and MMSE combiners in Equation (4) requires a complexity of $O(M^{3})$ due to the inversion of a matrix with size *M*. Hence, the application of the MF combiner can be a computationally efficient way to recover simultaneously transmitted 2D elemental images when a large number of sensors (or, equivalently, transmit antennas) are deployed in the wireless 3D image transmission system over a MIMO channel.

**Figure 6:**
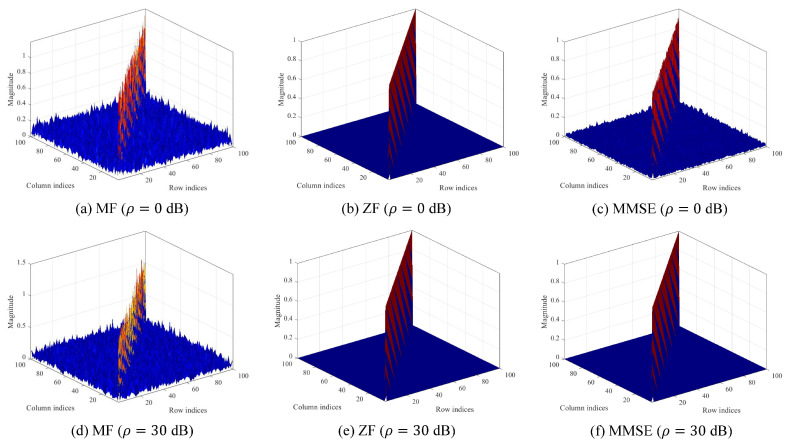
Magnitude plot of the combined channel G in Equation (5).

Figure 6 shows the magnitude plot of the combined channel G when M=N=100. From the numerical results in Figure 6, we discuss the effect of linear receivers by comparing a main diagonal entry and off-diagonal entries, which correspond to the magnitudes of a desired channel and interference channels, respectively. Because the main diagonal entry is significantly larger than the off-diagonal entries, we note that the application of the linear combiners can mitigate inter-stream interference effectively. Specifically, the results of Figure 6a,d reveal that the MF can mitigate inter-stream interference because the combined channel G tends to be diagonalized for low- and high-operating SNRs, respectively [28,29]. Figure 6b,e show that the ZF is capable of eliminating inter-stream interference by forcing the combined channel G to be $I_{N}$, regardless of the operating SNRs. Figure 6c,f indicate that the MMSE behaves similarly to the MF for a low-operating SNR (ρ=0 dB), while the combined channel is nearly diagonalized because the combining matrix of the MMSE approaches that of the ZF for a high-operating SNR (ρ=30 dB).

**Figure 7:**
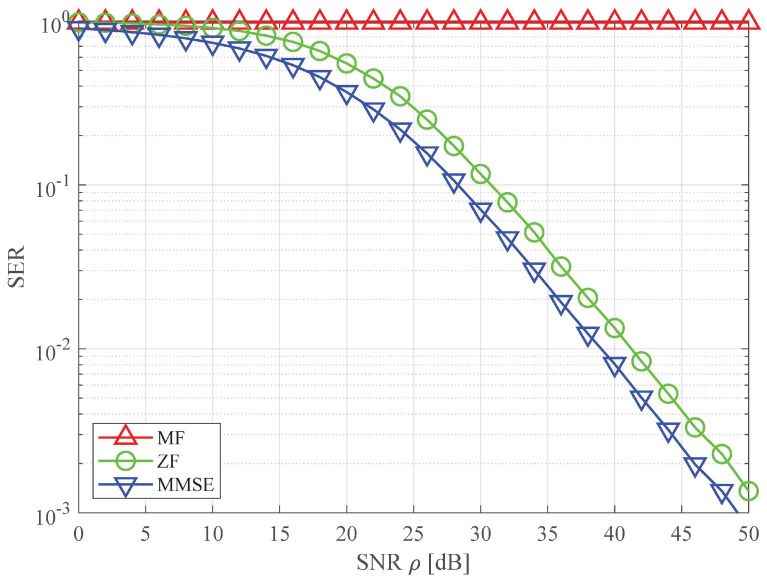
SER comparison of linear combiners over 100×100 MIMO systems with 256-QAM.

In Figure 7, we evaluate the symbol error rates (SERs) of three linear combiners based on a Monte Carlo simulation. Here, the SER is defined as a probability that an estimated symbol ${\hat{x}}_{m}$ is not equal to a transmitted symbol $x_{m}$. Figure 7 shows that the SERs of three linear receivers decrease as the operating SNR ρ increases. As is well known in the area of wireless communication [21,28], the MMSE achieves superior performance while the MF shows poor performance. The ZF shows competitive performance though the SER is slightly degraded with respect to that of the MMSE. In particular, it is worth noting from Figure 7 that the SER of the MF is nearly one for all operating SNRs. In other words, assuming one pixel corresponds to eight bits and is modulated to a 256-QAM symbol, most pixels composing an 2D elemental image are erroneously recovered at the receiver with high probability. The numerical analysis reveals that the MF may not be properly combining for the successful transmission of 2D elemental images from the perspective of wireless communication performance. However, in the subsequent section, the experimental results show that the application of the MF combiner is capable of achieving sufficient 3D reconstruction performance for the integral imaging in the wireless multisensor image system.

## 4. Experimental Results

In this section, we present our experimental setup and results to prove the feasibility of the proposed 3D wireless transmission system. In the experiment, we used four different 3D objects with different positions to obtain the elemental images by the SAII. In addition, to generate 3D digital contents, we used the VCR with non-uniform shifting pixels [11]. For the analysis over a MIMO channel, we assumed that 100 sensors capture 2D elemental images at the transmitter, and the receiver reconstructs 3D image. Considering the transmitter and receiver are equipped with 100 antennas (M=N=100), we simulated the 3D image transmission and reception over a flat Rayleigh fading channel with three operating SNRs (0 dB, 15dB, and 30 dB).

### 4.1. Experimental Setup

**Figure 8:**
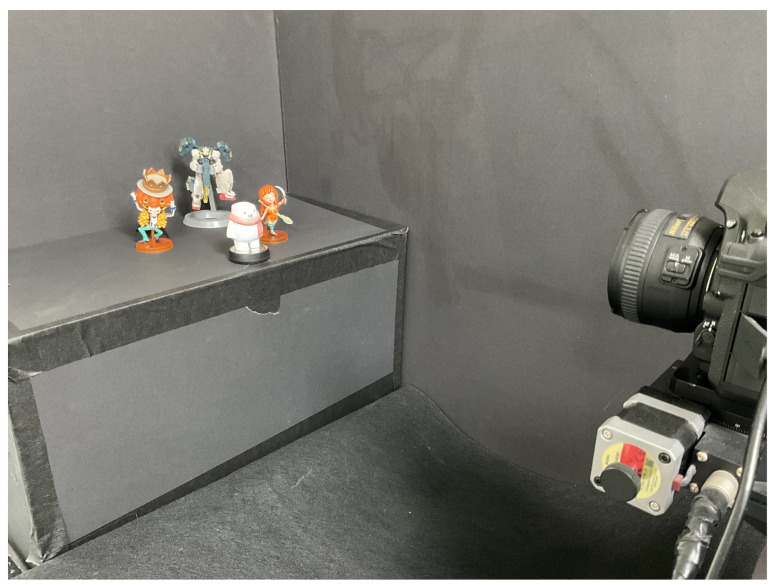
Experimental setup.

To obtain the elemental images of 3D objects, we used SAII in the pickup process as depicted in Figure 8. Four different 3D objects (white snowman, orange woman with sword, skeleton with pumpkin hat, and robot toy) are placed at different positions (352 mm, 368 mm, 420 mm, and 431 mm, respectively). The elemental image set consists of 10 (H) × 10 (V) elemental images with 1920 (H) × 1276 (V) resolution. In the SAII, the focal length of the camera (*f*) is 50 mm, pitch between cameras (*p*) is 2 mm in the *x* and *y* directions, and the sensor size is 36 mm (H) × 24 mm (V).

After the SAII captures 100 elemental images, those were transformed to QAM symbols with a modulation order 256 (Q=256), and transmitted simultaneously through the corresponding transmit antennas. After combining a received vector with one of the linear combiners in Equation (4), the receiver demodulated each transmitted symbol by applying the ML detection in Equation (7). By converting the demodulated symbols, the receiver was capable of acquiring 100 elemental images.

**Figure 9:**
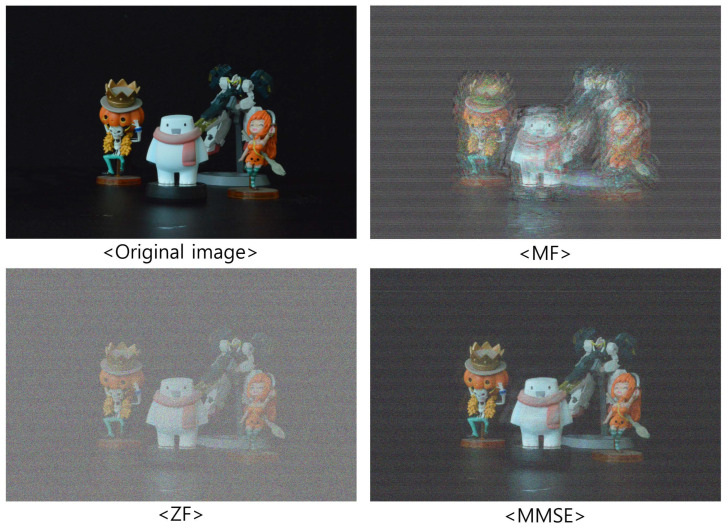
Elemental images received via different linear combiners when ρ=0 dB.

**Figure 10:**
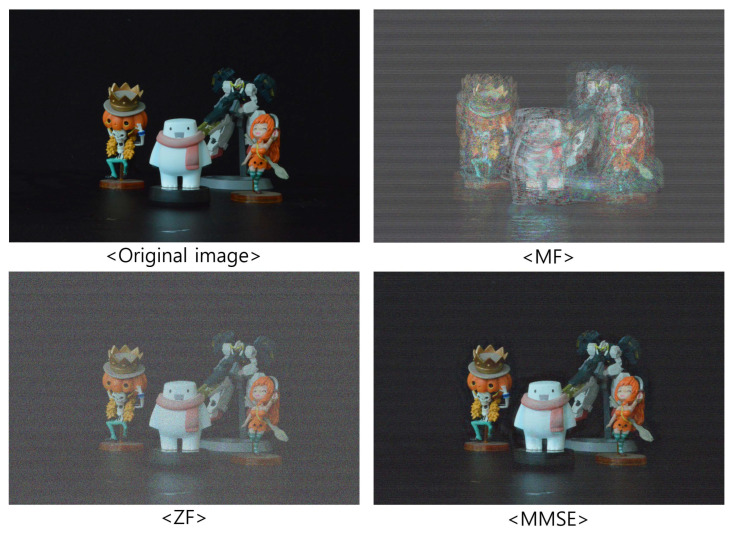
Elemental images received via different linear combiners when ρ=15 dB.

**Figure 11:**
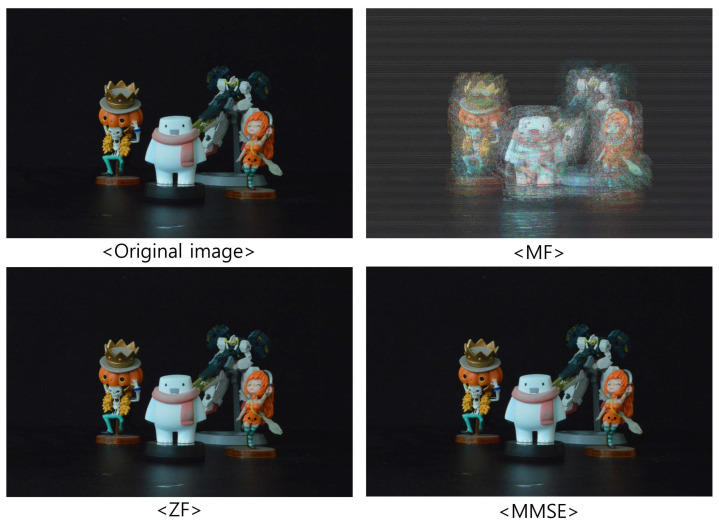
Elemental images received via different linear combiners when ρ=30 dB.

Figure 9, Figure 10 and Figure 11 show the received 2D elemental images obtained by different linear receivers when the operating SNR was set to 0 dB, 15 dB, and 30 dB, respectively. As shown in Figure 9, Figure 10 and Figure 11, the quality of the received elemental images depends on not only the operating SNRs but also the applied linear combiners. As expected from the results in Figure 7, the application of the MMSE combiner shows the best detection quality for all operating SNRs since it is the optimal linear combiner which achieves the maximum SINR. When the ZF combiner is used, the detected elemental images seem fairly noisy, in particular for a low SNR (i.e., ρ=0 dB), because of the noise boosting effect. Furthermore, we observe that the MF combiner shows the worst detection quality since the received elemental image is severely degraded by the remaining inter-stream interference, as anticipated from Figure 6 and Figure 7.

### 4.2. Experimental Results

**Figure 12:**
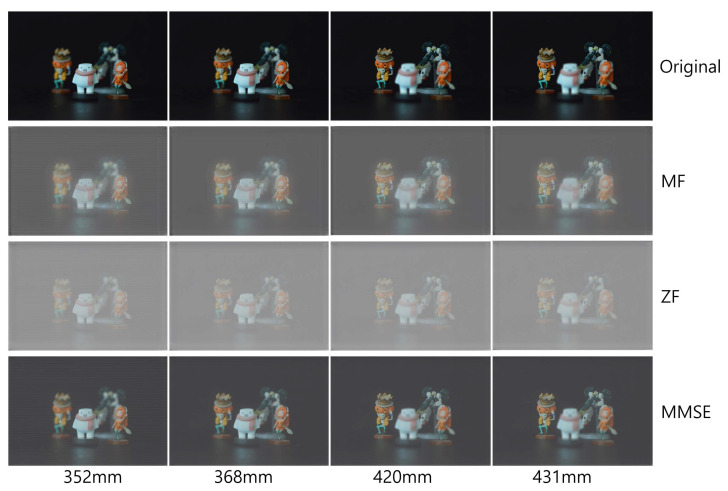
3D images reconstructed at various depths for different linear combiners when ρ=0 dB.

**Figure 13:**
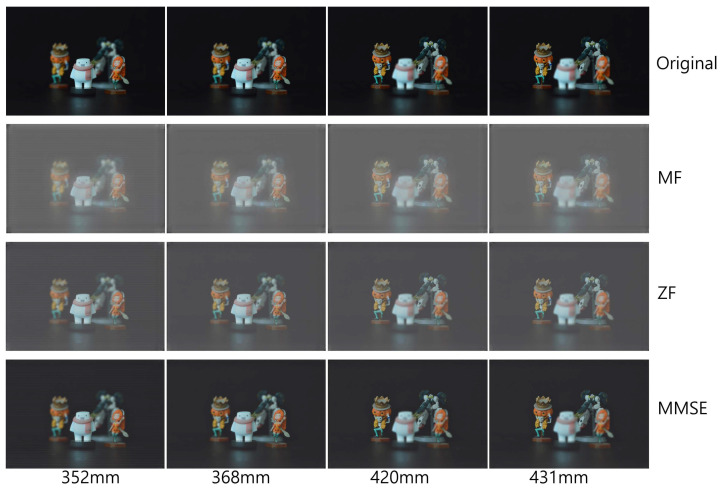
3D images reconstructed at various depths for different linear combiners when ρ=15 dB.

**Figure 14:**
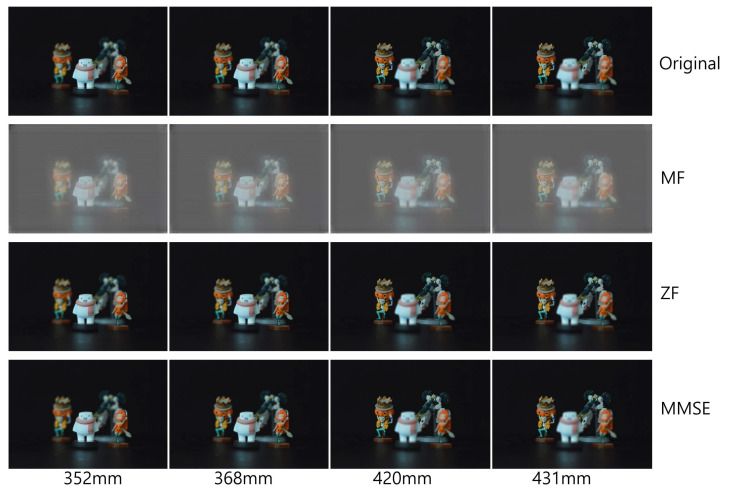
3D images reconstructed at various depths for different linear combiners when ρ=30 dB.

To reconstruct 3D images with the received elemental images, which are passed over the wireless MIMO channel, in this paper, we used the VCR with non-uniform shifting pixels. Although the received elemental images may be corrupted by not only the wireless channel but also inter-stream interference, these effects are expected to be mitigated by the VCR owing to the averaging effect. In addition, the VCR with non-uniform shifting pixels can provide more accurate reconstruction depths. Using Equations (1) and (2), 3D images at various depths are reconstructed with the received elemental images. In Figure 12, Figure 13 and Figure 14, the 3D reconstruction results show that the targeted 3D object is focused while others are out of focus. The results reveal that the VCR can effectively suppress the effects of the wireless channel and inter-stream interference inherent in the received 2D elemental images for all linear combiners. In particular, it is worth noting that the application of the MF combiner can achieve acceptable 3D reconstruction results with the aid of the VCR, in contrast to the severely degraded wireless communication performance in Figure 7, Figure 8, Figure 9, Figure 10 and Figure 11.

**Figure 15:**
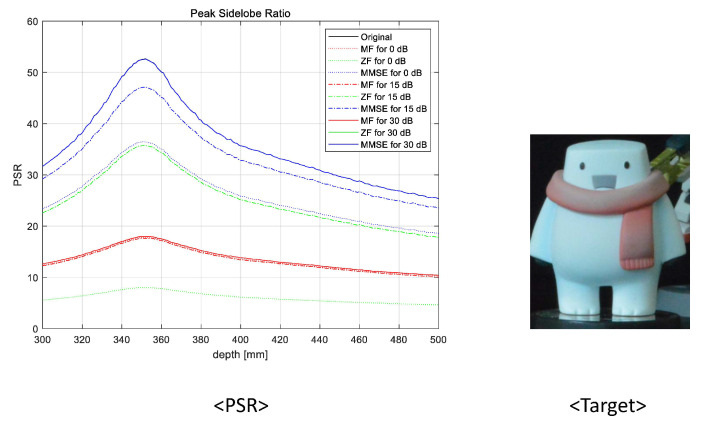
Peak sidelobe ratio for the first object at 352 mm.

**Figure 16:**
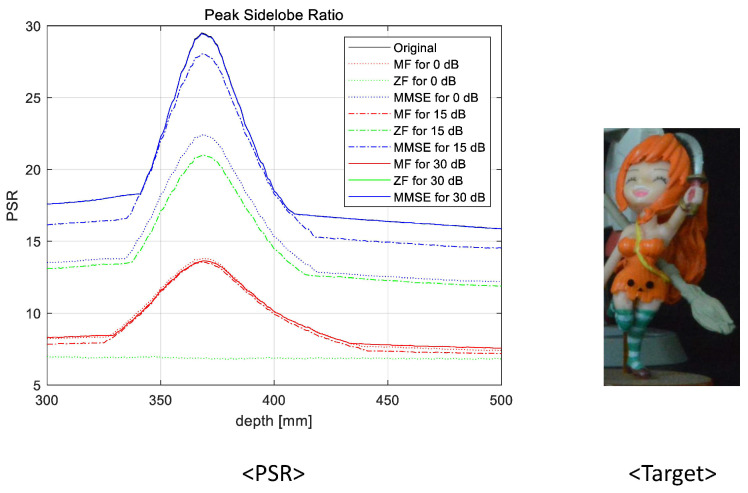
Peak sidelobe ratio for the second object at 368 mm.

**Figure 17:**
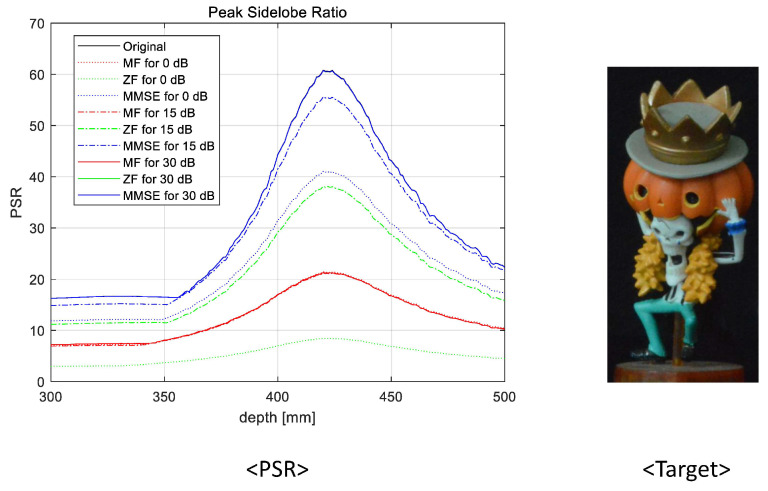
Peak sidelobe ratio for the third object at 420 mm.

**Figure 18:**
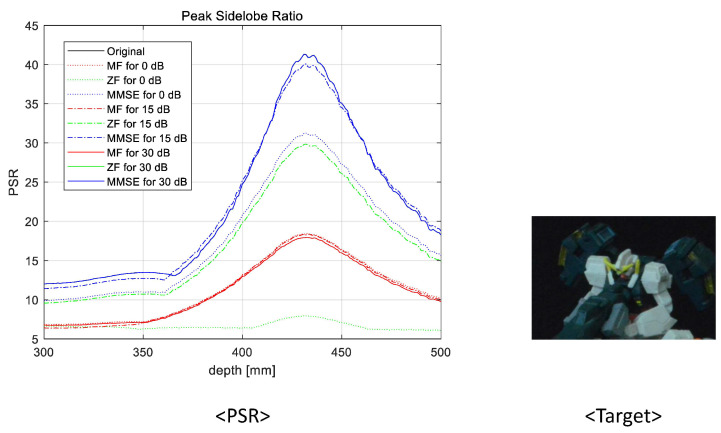
Peak sidelobe ratio for the fourth object at 431 mm.

To prove the feasibility of the proposed system, we reconstructed 3D images at various reconstruction depths, implemented non-linear correlation [30], and calculated the peak sidelobe ratio (PSR). The non-linear correlation is defined as

(8) $$c{\left(x,y\right)}_{z_{r}}={\left|F^{-1}\left[{\left(|I_{z_{r}}\left(\xi ,\eta \right)||R\left(\xi ,\eta \right)|\right)}^{k}e^{j(\theta_{I}-\theta_{R})}\right]\right|}^{2}$$

where $F^{-1}$ is the inverse Fourier transform, $I_{z_{r}}\left(\xi ,\eta \right)$ is the Fourier transform of the reconstructed 3D image at $z_{r}$, R(ξ,η) is the Fourier transform of the reference object image, $\theta_{I}$ is the phase of $I_{z_{r}}\left(\xi ,\eta \right)$, $\theta_{R}$ is the phase of R(ξ,η), and *k* is a non-linearity factor for correlation. In this experiment, four object images were used as the reference object images and the non-linearity factor is k=0.7. Then, the PSR can be calculated by using the following [31]

(9) $$PSR=\frac{c_{max}-\bar{c}}{\sigma_{c}}$$

where $c_{max}$ is the maximum value, $\bar{c}$ is the average, and $\sigma_{c}$ is the standard deviation of the correlation. In Figure 15, Figure 16, Figure 17 and Figure 18, we numerically analyze the PSR value, using Equations (8) and (9), for various reconstruction depths. The results validate that the highest PSR values are found accurately at the corresponding position of the desired 3D object for all operating SNRs and applied linear combiners. Therefore, we confirm that the proposed wireless 3D transmission system can transmit 3D digital content through a wireless MIMO channel effectively. Among the three combiners, the MMSE combiner achieves the highest PSR regardless of the operating SNRs and reconstruction depth. Although the ZF combiner shows excellent performance for mid- and high-operating SNRs (i.e., 15 dB and 30 dB, respectively), the PSR performance is degraded with respect to that of the MF for a low-operating SNR (i.e, 0 dB) due to the noise boosting effect. In particular, despite the poor wireless communication performance, we note that the MF is also an eligible combining technique because a reasonable PSR value can be achieved while using the lowest computational complexity.

## 5. Conclusions

In this paper, we have considered 3D image transmission of integral imaging through a wireless MIMO channel. Due to the large number of elemental images required in integral imaging for 3D image reconstruction, we need a 3D image transmission technique able to support a high transmission rate. Accordingly, we proposed to transmit and receive a number of elemental images using the MIMO technique in order to reconstruct 3D images having reasonable visual quality via the VCR with non-uniform shifting pixels. The experimental results validated that the proposed techniques are capable of achieving excellent performance for the 3D image reconstruction. Specifically, the numerical analysis revealed that the MMSE combiner achieves the best performance for various operating SNRs and the depths of 3D objects, whereas the ZF combiner may be vulnerable to the AWGN because of the noise boosting effect. Despite the severely deteriorated wireless communication performance, the MF shows promising performance, with the lowest computational complexity owing to the averaging effect of the VCR. Nevertheless, as future work it would be worthwhile to improve the performance of the proposed system applied with the MF combiner in order to overcome the performance gap with the optimal combiner, i.e., MMSE. By deploying multiple antennas at the transceiver, we expect that the proposed system can efficiently convey 3D digital content for various applications such as AR, VR, metaverse, 3D TV, etc.

## Data Availability

Not applicable.

## Abbreviations

The following abbreviations are used in this manuscript:

2D	Two-dimensional
3D	Three-dimensional
AR	Augmented reality
AWGN	Additive white Gaussian noise
CSI	Channel state information
MALT	Moving array lenslet technique
MF	Matched filter
MIMO	Multiple-input multiple-output
ML	Maximum likelihood
MMSE	Minimum mean squared error
PSR	Peak sidelobe ratio
QAM	Quadrature amplitude modulation
SAII	Synthetic aperture integral imaging
SINR	Signal-to-interference-plus-noise ratio
SNR	Signal-to-noise ratio
V-BLAST	Vertical Bell Laboratories layered space–time
VCR	Volumetric computational reconstruction
VR	Virtual reality
ZF	Zero forcing
